# FieldNA: a 3D printed vertical microfluidic device for portable nucleic acid isolation from olive oil samples

**DOI:** 10.3389/fbioe.2025.1646041

**Published:** 2025-10-13

**Authors:** Vineet Penumarthy, Athanasia-Maria Dourou, Evangelia Lampropoulou, Stilianos Arhondakis, Ravi Prakash

**Affiliations:** ^1^ Department of Electronics Engineering, Carleton University, Ottawa, ON, Canada; ^2^ BioCoS P.C., Chania, Crete, Greece

**Keywords:** portable DNA isolation, 3D printed microfluidics, magnetic beads, extra virgin olive oil (EVOO), real-time PCR, high resolution melt (HRM) analysis

## Abstract

Isolation and purification of nucleic acid is an essential step in molecular assays for several application areas including healthcare, food safety and security, environmental monitoring, forensic science, etc. Nucleic acid extraction is a critical bottleneck towards field deployable nucleic acid-based assays, limiting them to laboratory setups and bench-top configurations. In addition, this lack of portability leads to longer timelines for sample processing and time-to-results, and higher testing costs, limiting access to this highly sensitivity assay tool in many instances. Several efforts have explored the creation of portable nucleic acid extraction systems to complement recent innovations in reducing the footprint and overhead of the nucleic acid test assays; however, most solutions are dependent on supporting systems such as power supply, and peripheral laboratory equipment (centrifuges, incubators). In this work, we present a novel 3D printed and fully disposable device, FieldNA, which minimizes specialized reagents and laboratory equipment requirements for nucleic acid extraction. The device relies on gravity driven vertical flow, and a magnet assisted bead washing and solid-liquid separation phase. Its functionality is demonstrated through DNA extraction from olive oil samples, and its performance is compared to three widely used extraction methods: CTAB combined with phenol chloroform (PCl), and two commercial filter column-based DNA extraction kits. The optimized FieldNA device prototype repeatably produced nucleic acid yield and quality comparable to the above lab-based olive oil DNA extraction techniques. The 3D printed device’s performance in isolating olive DNA from different batches of olive oil samples indicates its suitability for handling complex agricultural products, and the viable scalability for implementation in a wide spectrum of applications ranging from food to health sector.

## 1 Introduction

Extra virgin olive oil (EVOO) holds a significant place in the market due to its health benefits, attractive qualities, and limited production (D’Adamo et al., 2019; Jimenez-Lopez et al., 2020; Chrysochou et al., 2022; Latino et al., 2022). As a premium product with high value, it has long been a primary target for fraud. Therefore, having a certification label of DNA verified, and access to technologies for assessing its varietal composition, is essential to combat food fraud, ensure consumer’s correspondence between label and content, and trace the oil’s journey from “field to bottle.” The European Commission has set regulations to establish and ensure quality standards for agricultural products and labelling restrictions for food items (*Regulation (EU) 2024/1143*). However, EVOO authentication and traceability has emerged as a critical requirement (Conte et al., 2020) to prevent intentional mislabeling or adulteration through the deliberate mixing of low-cost, edible vegetable oils (Sudhakar et al., 2023).

In recent years, DNA-based methods have provided improved accuracy and reliability over chemical authentication techniques, as they are unaffected by environmental conditions (Salah and Nofal, 2021; Hashempour-baltork et al., 2024; Mascio et al., 2024). Among the various genetic markers for identifying cultivars for the authentication of processed plant oils such as olive oil (OO), simple sequence repeats (SSRs) and single nucleotide polymorphisms (SNPs) have proven to be the most effective (Ben-Ayed et al., 2012; Gomes et al., 2018; Carvalho et al., 2021; Xiao et al., 2024). A key requirement for this method is obtaining sufficient high-quality DNA from olive oil for the downstream analysis. Several studies have explored different OO extraction protocols utilizing different organic solvents (e.g., n-hexane, octane, etc.) as their initial step, followed by DNA recovery and precipitation and lastly its purification. Some of these protocols utilize reagents such as β-mercaptoethanol, EDTA and/or SDS, CTAB (cetyltrimethylammonium bromide). However, despite the availability of various DNA-based approaches for the authentication and traceability of OOs, significant challenges remain in standardizing the DNA extraction process, and the subsequent amplification and genotyping stages.

Commercial DNA extraction platforms provide high-purity DNA yields but require skilled personnel, expensive lab-based equipment, and are unsuitable for field use, limiting the development of point-of-care testing (POCT) devices for DNA authenticity in the field. Spin-column methods (e.g., Qiagen-QIAamp) require around 60 s per centrifugation step, but the total process takes 30–60 min per sample due to incubation and reagent handling, with sample volume requirements ranging from 200 μL to 2 mL (Kibar et al., 2024). Magnetic bead-based methods offer a faster, more cost-effective alternative while relying on similar solid-phase extraction principles (Huang et al., 2023; Kibar et al., 2024). More recently, microfluidic DNA extraction systems have emerged, offering miniaturized platforms that operate at µL or nL scales with improved accuracy, shorter processing times, and reduced contamination risk (Toldrà et al., 2023; Kibar et al., 2024). These systems have evolved into various formats, including cartridges, Lab-on-a-disc devices, paper-based platforms, and PDMS microchips, thereby broadening their use for rapid, on-site testing in non-laboratory settings (Huang et al., 2023; Schlenker et al., 2023; Toldrà et al., 2023; Zhong et al., 2023; Kibar et al., 2024; Nguyen et al., 2024). Such microfluidic DNA extraction processes have integrated precisely controlled fluid flow with surface modified magnetic beads that are influenced by a magnetic field created using electromagnets, integrated soft magnets, or external permanent magnets (Azimi et al., 2011). The manipulation of magnetic beads may be classified as 1) immobilized beads or static bead chains 2) temporarily immobilized beads and 3) transported beads. In the first two classes, a magnetic field captures the magnetic beads and either holds them permanently in a specific location in the microchannel as in the first group or captures them in a location for a specific amount of time and re-suspends them in fluid following a certain amount of time, as in the second group. The third group is facilitated by a moving magnetic field without stopping. However, successful bead transport is highly sensitive and depends on various factors such as flow rate of reagents, magnetic field strength, immobilized surface orientation and material properties, etc.

In the present study, we propose FieldNA, a novel 3D-printed magnetic DNA isolation device, which uses a series of stacked modules to direct fluid flow vertically and into a magnetic bead capture module where magnetic beads are captured and immobilized on an incline plane coated with a specialized polymer membrane. Once immobilized, various buffers are passed over the magnetic beads to perform the washing and the DNA elution steps, where the purified DNA is captured in a separate plate module. This purified DNA can now be used for further downstream applications using a wide range of molecular analyzers. The herein device was implemented to perform binding, washing and elution steps to isolate DNA from different olive oil samples within 20 min with an initial olive oil sample volume of 500 μL, without the need for any sophisticated lab hardware or external support resources (e.g., power, running water). The quality of extracted DNA was established using spectrophotometry and real-time PCR analysis and the FieldNA performance was compared to three established and widely used DNA extraction methods. The findings of this study prove the suitability of this new device as a viable alternative method to isolate DNA from olive oil and other matrices, and in combination with portable real-time -PCR platforms, it could provide a rapid, reliable, and on-site testing method at low cost with high scalability potential for a wider range of field-based applications beyond olive oil.

## 2 Materials and methods

### 2.1 Device design, fabrication, and operation

**FIGURE 1:**
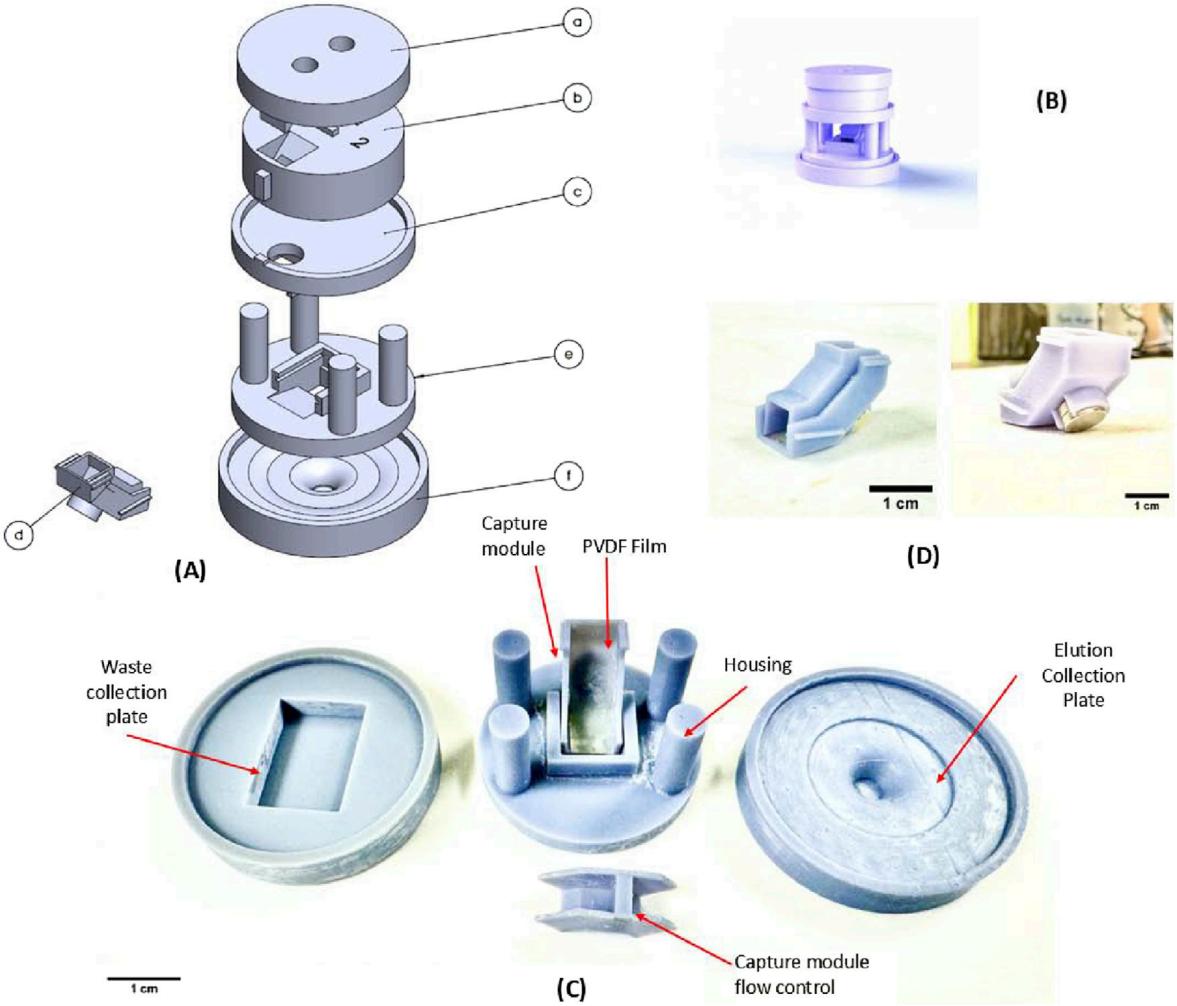
3D rendering of the assembly of the device and the 3D printed device components. **(A)** The components are as follows: (a) Input module: top plate (b) Input module: Mating plate (c) Magnetic separation plate (d) Magnetic capture module (e) Base housing plate (f) Elution Collection plate. **(B)** 3D rendering of the assembled device **(C)** Magnetic capture module in use. **(D)** Fully assembled magnetic bead capture module.

The salient feature of the FieldNA device is the vertical stacking of the various fluidic modules, a collection of components designed to perform DNA isolation, as shown in Figures 1A,B. The topmost module consists of components a and b, which allows for sample loading and an incubation period. Through aligned holes and incline profiles, fluidic sample is regulated downwards in the separation plate (component c) and then towards the magnetic capture module (component d; Figures 1A,C,D), which is aligned and supported using a housing structure (component e). The purified DNA is collected in the bottom elution plate, component f, which again consists of a unique profile allowing for easy transfer of eluted DNA. The main goal for the device design is to facilitate gravity-driven fluid flow, with flow control implemented at various stages using modular components. Several optimization steps were implemented in printing of individual modules, most notably the magnetic capture module shown in Figures 1C,D. It consists of two components: a bottom surface with a 45^o^ inclined flow profile with an *in-house* synthesized polyvinylidene fluoride (PVDF) membrane, and a top enclosure with a built-in sluice gate-like flow control structure to control the speed and alignment of fluid flow over a Neodymium disc magnet (Diameter: 6 mm, height: 3 mm, Applied Magnets). Additional modules may be stacked above or below the current setup, depending on unique target application requirements.

The device was designed in SolidWorks 2022, and the reported prototype has a total footprint of approximately 52 mm × 52 mm with a height of 50 mm. In the outlet of component b in Figure 1, there is a PDMS circular insert to facilitate flow focusing and to reduce dead volume. The magnetic bead capture module is designed to be a single-use disposable unit which may be replaced following an extraction experiment. The design was processed for stereolithography (SLA) 3D printing using CHITUBOX (v1.9.5) with the following settings: exposure time: 10 s, lift distance: 7 mm, lift speed: 90 mm/min, bottom exposure time: 50 s, layer height: 0.050 mm, retract speed: 320 mm/min. Finally, printing was carried out using a Elegoo (Shenzen, China) Saturn 3 Ultra 3D printer, with Grey ABS-like resin UV Resin, as the printing material. The parts were post-processed in an isopropyl alcohol (IPA) bath followed by 10 min of further UV curing using the Elegoo Mercury X wash and curing stations. All components were coated in a Teflon coating (4,5-Difluoro-2,2-bis(trifluoromethyl)-1,3-dioxole, polymer with Tetrafluoroethylene).

Once the user loads the solution containing lysate media and bead-bound DNA into the device through the top module (a and b), the solution is retained for a desired incubation period. Following this, the content is driven downwards into the magnetic capture module by aligning the notch features in module b and c, achieved through rotation. This allows the solution to enter capture module d, where it flows over the inclined PVDF film covering the disc magnet. The magnet housed in the bottom structure captures the beads and with help from the control structure, the beads are immobilized uniformly on the PVDF film. The remaining solution passes through flowing downwards into a waste collection plate, used to collect all discard lysis and wash buffers. The magnetic beads, now immobilized, are in place for the washing, drying and elution steps. For the final step, when the elution media is added to detach purified DNA from beads, the waste collection plate is swapped with the elution plate mentioned above.

### 2.2 Plant material and olive oil used in DNA isolation experiments

Olive leaf and olive oil samples were provided to BioCoS by Aprol Umbria (Umbria, Italy), including different olive varieties. Olive oil samples used in this study corresponded mainly to 2024 harvest year (EVOO-24), with a handful of experiments utilizing samples from 2023 harvest year (EVOO-23). The isolated DNA from olive leaf samples served as a positive control for the real-time PCR, coupled with High Resolution Melting (HRM) analysis. In this study, the Italian cultivar “Moraiolo” was utilized for both olive leaf and olive oil DNA isolation experiments. Due to the limited availability and the observed poor DNA quality in the older batch (EVOO-23), the experimental results and statistical analysis presented in the main manuscript text correspond to the EVOO-24 samples. Results obtained from EVOO-23 samples are reported separately in Supplementary attachment 2.

### 2.3 DNA isolation from olive leaf samples

Genomic DNA was extracted from cv. Moraiolo young olive leaf tissue using a modified CTAB method (Cipriani et al., 2002), which is found to be well-suited for plants with high polysaccharide and polyphenol content. 200 mg of fresh olive leaves were frozen in liquid nitrogen and ground to a fine powder. The ground tissue was mixed with 1 mL of pre-warmed (at 65 °C) CTAB extraction buffer (2% CTAB, 1.4 M NaCl, 100 mM Tris-HCl pH 8.0, 20 mM EDTA, 0.2% β-mercaptoethanol) and incubated at 65 °C for 30 min to facilitate cell lysis, followed by centrifugation for 15 min at 14000rpm at 4 °C. After the recovery of the clean lysate into a new tube, an equal volume of chloroform:isoamyl alcohol (24:1) was added, and the mixture was centrifuged for 15 min at 14000rpm at 4 °C. The aqueous phase containing DNA was transferred to a new tube, and DNA was precipitated with 100% ice-cold isopropanol at −20 °C for 30 min. After centrifugation for 20 min at 14000rpm at 4 °C, the DNA pellet was washed twice with 70% ethanol and air-dried. The DNA pellet was finally resuspended in 50uL of TE buffer (10 mM Tris, 1 mM EDTA, pH 8.0) and stored at −20 °C until further analysis.

### 2.4 DNA isolation protocols implemented for olive oil samples

**FIGURE 2:**
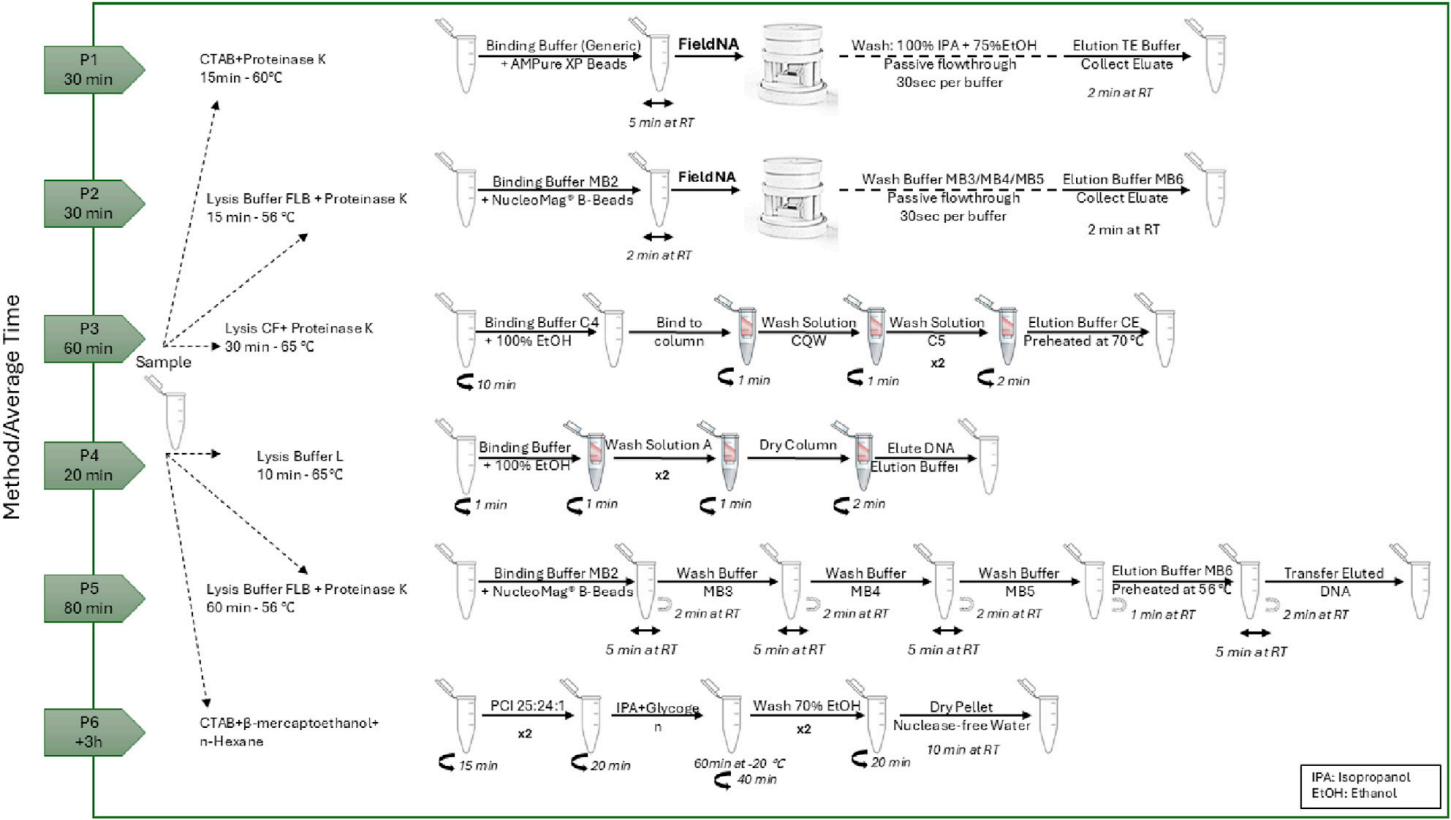
Schematic representation of the DNA isolation protocols and their average time to completion.

Six different DNA isolation protocols for Olive Oil were implemented in this work. All samples were extracted in quadruplicate (n = 4) using each protocol, and the yield and purity of the extracted DNA was measured using BioPhotometer D30 (Eppendorf). Figure 2 presents a detailed process flow diagram for each of the used protocols.

#### 2.4.1 DNA isolation using FieldNA

Genomic DNA was isolated from olive oil using the FieldNA device with two distinct protocols: i) generic CTAB-based protocol, using simple chemistry (P1) and ii) NucleoMag DNA Forensic Kit (MACHEREY-NAGEL, Düren, Nordrhein-Westfalen, Germany) chemistry (P2).

In the P1 protocol, 500 μL of olive oil cv. Moraiolo was added in a 2 mL centrifuge tube and 600 μL of CTAB buffer (ready to use), and 30 μL of Proteinase K–all provided by Promega Corporation (Madison, WI, United States) – were added to the sample. This step was followed by an incubation at 65 °C for 15 min, inverting the samples every 5 min. The clear lysate (lower phase) was recovered carefully without the use of a centrifuge and transferred to a new 1.5 mL tube. 300 μL of Binding Buffer (5 M guanidine hydrochloride, 40% (vol/vol) Isopropanol, 0.12 M sodium acetate and 0.05% (vol/vol) Tween 20), and 30 μL of AMPure XP Beads for DNA Cleanup (Beckman Coulter, Inc.) were added. The sample was mixed gently for 5 min and then added into FieldNA, where beads were immobilized on the PVDF film. Two washing steps were followed, one with 500 μL of 100% Isopropanol and another with 600 μL of 75% ethanol. The cartridge was removed from the device and left to dry for 2 min. After reinsertion of the cartridge back to FieldNA, the elution was carried out using 40 μL of TE (10 mM Tris, 1 mM EDTA, pH 8.0) buffer. The purified DNA was collected from the plate and then used for further downstream analysis.

In P2 protocol, lysis was initiated by adding 300 µL of Lysis Buffer FLB and 20 µL of Proteinase K solution from the NucleoMag DNA Forensic Kit (MACHEREY-NAGEL, Düren, Nordrhein-Westfalen, Germany) into a 1.5 mL centrifuge tube containing an initial olive oil sample volume of 500 μL, followed by thorough mixing and incubation at 56 °C for 15 min. After incubation, two distinct phases were formed: an upper oily phase and a lower aqueous phase. The lower phase was recovered and added to a new 1.5 mL tube. DNA binding to magnetic beads was facilitated by adding 340 µL of Binding Buffer MB2 and 14 µL of resuspended NucleoMag^®^ B-Beads to the lysate and then mixing gently for 10 times. The solution was then added to FieldNA, leading to bead immobilization on the PVDF film. This was followed by washing steps beginning with 600 µL of Wash Buffer MB3 added to the device, and 600 µL of Wash Buffer MB4. A final wash was performed using 900 µL of MB5. The module was then removed from the device and inverted to allow for drying for 2 min at room temperature before being reinserted. Finally, the purified DNA was eluted by first changing the plate underneath to the collection plate and then adding 40 μL Elution Buffer MB6. The purified DNA was collected from the plate and then used for further downstream analysis.

#### 2.4.2 DNA isolation using commercial kits and CTAB/PCI method

Three commercial kits and one variation of a CTAB-based method were employed to verify the FieldNA device performance with P1 and P2 protocols. Total DNA was isolated and purified from 500 µL of olive oil using i) the NucleoSpin Food Kit (MACHEREY-NAGEL, Düren, Nordrhein-Westfalen, Germany) (P3) following the manufacturer’s instructions, ii) the Olive Oil DNA Isolation Kit (Norgen Biotek Corp, Thorold, Ontario, Canada) (P4) following the manufacturer’s instructions, iii) NucleoMag DNA Forensic Kit (MACHEREY-NAGEL, Düren, Nordrhein-Westfalen, Germany) (P5) following the manufacturer’s instructions and iv) a modified CTAB protocol (P6), to achieve a final elution volume of 40 μL in each experiment. Briefly, for P6, the protocol modifications were as follows: 500 μL of olive oil was transferred into a 2 mL centrifuge tube and mixed with 350 μL of ice-cold n-hexane. Extraction buffer 1 mL (998 μL CTAB buffer, ready to use provided by Promega Corporation, Madison, WI, United States, 2 μL β-mercaptoethanol) was added to and the sample was mixed thoroughly and incubated at 65 °C for 30 min. After letting the sample settle briefly, the tube was centrifuged for 15 min at 14000rpm. The aqueous phase was then transferred into a new 1.5 mL tube, mixed with 500 μL of phenol:chloroform:isoamylalcohol (25:24:1) (AppliChem GmbH Darmstadt, Germany), and then centrifuged for 20 min at 14000rpm. The aqueous phase was transferred to a new 1.5 mL tube, ice-cold isopropanol (0.8 volume) and 4 μL of glycogen (20 mg/mL) were added. The mixture was gently mixed and incubated for 60 min at −20 °C. Following centrifugation (40 min at 14000rpm), the pellet was washed twice with 70% ethanol and dissolved in 40 μL of nuclease-free water. All centrifugation steps were carried out at 4 °C.

### 2.5 Real-time PCR set-up and high-resolution melt (HRM) analysis

A real-time quantitative PCR (qPCR) coupled with HRM software was carried out to determine the performance of the different extraction protocols. For this analysis, a publicly available Simple Sequence Repeat (SSR) genetic marker sequence, namely, ssrOeUA-DCA3 (F-CCCAAGCGGAGGTGTATATTGTTAC, R-TGCTTTTGTCGTGTTTGAGATGTTG), was synthesized and purchased from IDT (Integrated DNA Technologies, United States) and utilized as primer set for the real-time PCR reaction. For the reactions, the protocol described by Gomes et al., 2018 was employed, with minor modifications. More specifically, qPCR amplification of ssrOeUA-DCA3 was performed in a final volume of 20 μL, containing 10 µL 2X Forget-Me-Not™ EvaGreen^®^ qPCR Master Mix (Biotium CA, United States), 0.5 μM of each primer, and 50 ng of isolated DNA. All qPCR reactions were performed on a Mic qPCR Cycler (Bio Molecular Systems) using the following protocol: a 2-min incubation at 95 °C, followed by 40 cycles of denaturation at 95 °C for 5 s, an annealing step at 50 °C for 10 s, and a 12 s extension at 72 °C. For the melting phase, conditioning was done at 95 °C for 30 s and 60 °C for 60 s, leading to a melt analysis over the temperature range of 65 °C–95 °C, with increments of 0.2 °C/s, with constant green fluorescence signal acquisition.

A 2% agarose gel electrophoresis was set up and the real-time PCR products were run at 50V for 60 min to confirm that the amplification observed in the real-time PCR matched the expected amplicon size, in comparison to the positive control (Supplementary Figure S1.1).

### 2.6 Statistical analysis

All DNA isolations were conducted in quadruplicates per protocol to capture inherent sample-to-sample variability and to ensure statistical robustness of the measurements. This approach increases the accuracy of variance estimation, reduces the influence of outliers, and provides improved power for subsequent statistical analyses, while maintaining experimental feasibility. To assess potential variability into the DNA isolations, two operators were involved, each having different laboratory experience (one is a senior scientist and the other a junior scientist). This way we can also address the user-friendliness of the FieldNA. Data obtained from the photometer (DNA concentration and A_260/280_ ratio) were analyzed in R v 4.5.1 software using the car packages to assess homogeneity of variances with Levene’s test. Normality within groups was evaluated using the Shapiro–Wilk test implemented in the base stats package. Depending on the outcome of assumption checks, either a one-way ANOVA followed by Tukey’s HSD *post hoc* test, or a Kruskal–Wallis test followed by pairwise Wilcoxon comparisons with Holm correction, was performed. A significance threshold of α < 0.05 was applied for all statistical tests. All graphs were prepared with OriginPro, Version 2024b.

## 3 Results

### 3.1 Optimizing the magnetic phase separation

The FieldNA device is designed to integrate external cell lysis and incubation with magnetic bead-based isolation and purification into its workflow. After lysis and incubation, wash buffers containing specific reagents as described in the methods Section 2.3, can be introduced into the device’s input module for DNA purification on immobilized magnetic beads. The gravity-driven passive flow simplifies the device operation, as it requires no electrical power and ensures efficient transport of the sample to the capture module, optimizing the DNA isolation process. Other 3D printed platforms have incorporated equipment such as syringe pumps or used capillary forces, etc., to move fluid into and through their devices (Kibar et al., 2024; Kirkoyun Uysal et al., 2024; Holladay et al., 2025). It allows them to control flow rates more precisely and set specific flow rates, however complexity of the device increases.

**FIGURE 3:**
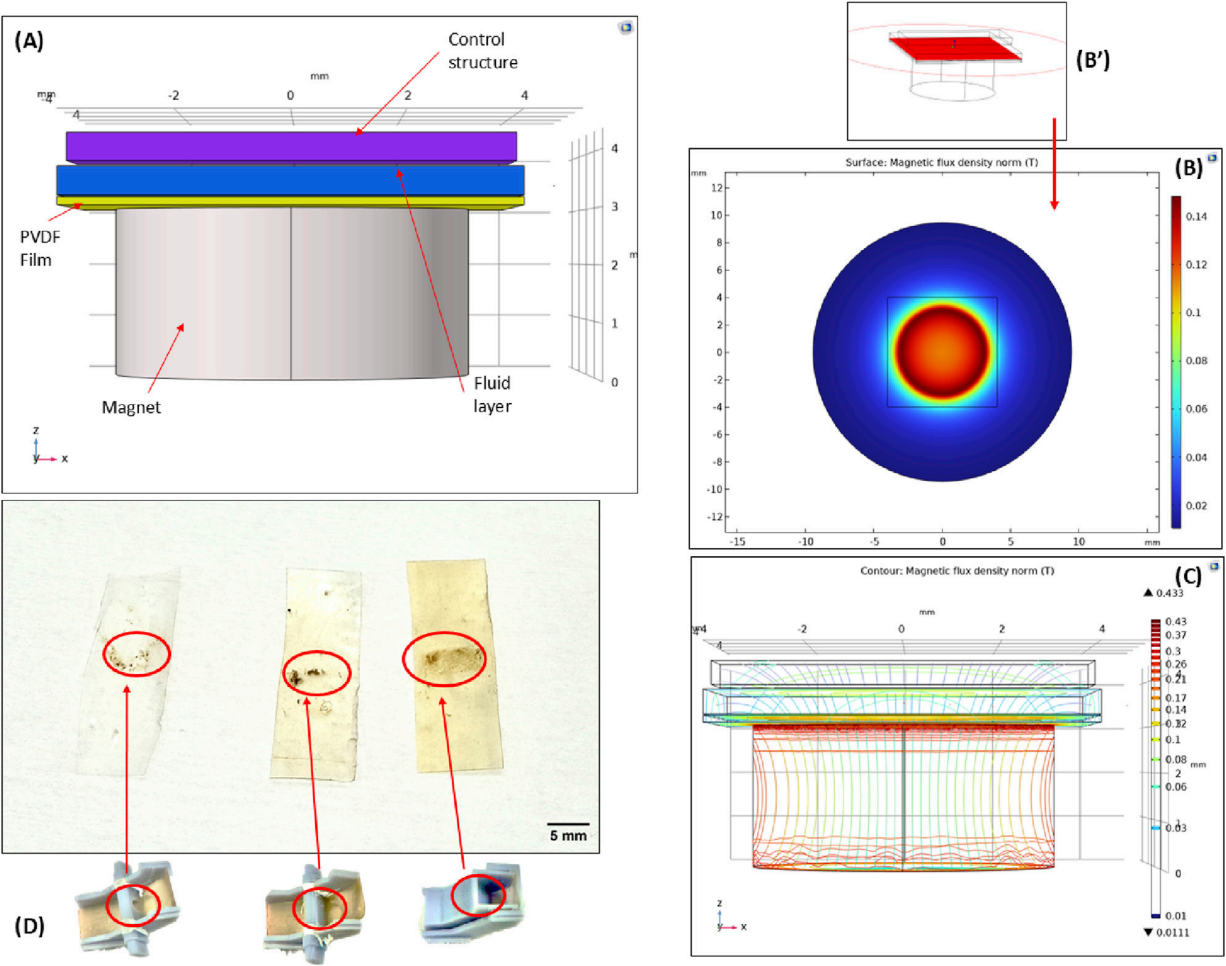
Magnetic bead-capture mechanism **(A)** The 3D schematic rendering of the region of capture **(B)** COMSOL simulation surface plot showing the magnetic flux density in the region. The entire device is enclosed in air. **(B’)** Cut plane location of surface plot. **(C)** Contour plot showing the magnetic flux density in the region of capture. **(D)** PVDF films with magnetic beads immobilized on the surface. The three films from left to right show the influence of the flow control structure during bead capture. The progression of the flow control profile can be seen corresponding to each film bead capture pattern.

Through flow validation tests, the average time taken for the fluid to flow into the magnetic capture module was determined to be within 1–2 min. Once in the capture module, the slant was designed to accommodate both the flow-through of the fluid, as well as to allow for the capture of the beads by the magnetic field. COMSOL Multiphysics simulation of the magnetic capture module design concept was conducted to verify the influence of the magnetic field in the capture module operation, as seen in Figure 3. Figure 3A shows the schematic rendition of the capture mechanism. The magnetic beads (diameter 1–3 μm) are located in the fluid layer as they pass through the capture module. The material of the surrounding area outside the cartridge was set to air with a boundary condition of magnetic insulation. Simulations were conducted using the magnetic fields, no current (mfnc) interface. The relevant parameters are relative permeability of permanent magnet 100, permanent magnet x axial remanence 0, permanent magnet y axial remanence 0, permanent magnet z axial remanence ±0.385 T. Figures 3B,C show the magnetic flux density distribution in this region, with Figure 3B’ denoting a cut plane in the fluid region. From literature, external magnetic fields ranging from 0.1 to 0.3 T are sufficient to influence and capture beads (Yadav et al., 2024; Dowling and Kostylev, 2025). From the surface plots, it is evident that the magnetic field permeates through the PVDF film, the thin polymer structural layer, and the fluid layer, with sufficient field strength to attract the beads in the fluid layer and capture them onto the PVDF membrane.

Following the initial proof-of-concept tests to demonstrate bead capture, a sluice gate-like control structure was designed to slow the flow of fluid and to facilitate a wider surface area for bead capture. As seen in the left-most film in Figure 3D, the proof-of-concept tests showed the feasibility of this process but resulted in a greater loss of sample and bead volumes. A flow control structure was introduced to combat this issue. Initially, this structure was designed as an external component inserted into the magnetic capture module (Figure 3D, centered figure), but it was later merged into the top component (Figure 3D, right-most figure). By introducing a large gate, the flow rate of the solution is reduced, and the fluid is concentrated over the magnet allowing for more capture time before passing through the module. This structure also serves another purpose in aiding with magnetic bead spread on the PVDF film. From the initial tests, the magnetic beads were captured on the film but lacked a uniform spread and tended to clump together. The structure was designed to increase the contact time of the beads with the film, enhancing surface interactions and resulting in greater immobilization and higher bead concentration. This was qualitatively validated for the various control structures based on the same amount of bead loading. The beads are spread horizontally widthwise across the film, aligned with the control structure. From left to right, the immobilized bead concentration increases due to optimizations in control structure design. It should be noted that the wash buffers have high concentrations of ethyl alcohol (ethanol). The behavior of this fluid is more unpredictable and harder to restrain (Srisomwat et al., 2024) so a wider spread in bead capture was desirable to allow for greater wash efficiency to accommodate for this variability in fluid flow. This was established through spectrophotometer characterization of the eluted DNA obtained from various prototypes designs with the flow control structures shown in Figure 3D.

Another motivation for the sluice gate control structure is improving the quality of the releases DNA. Purification is crucial for assuring the quality and yield of DNA. The control structure allows for a wider spread of the wash buffers as well as a lower flow rate for the elution buffer. The lower flow rate allows for longer elution buffer incubation time, mimicking the incubation step in commercial DNA extraction column kits where the elution buffer is deposited on the column and incubated at room temperature before centrifuging into an elution collection tube. By slowing down this flow through speed, the purification process has been optimized for this system.

### 3.2 Evaluation of the isolated DNA yield and purity using spectrometer

To evaluate the different protocols, we applied criteria including average DNA yield and purity (A_260/280_ ratio), with results presented in Table 1 as mean values from four replicates. In addition, a per-sample quantitative assessment of solvent use (liquid waste), plastic tubes, and pipette tips was performed, and these data are also summarized in Table 1.

**TABLE 1:** Performance comparison of six DNA isolation protocols. Reported values include average DNA yield and purity (A_260/280_), PCR amplification success, and practical parameters related to laboratory waste and consumables.

Protocol	DNA yield (ng/µL, mean)	Purity (A_260/280_, mean)	Positive PCR amplifications (n/total)	Waste volume (mL)	Consumables used (tubes/tips)
P1	47.3	1.8	3/4	3.0	3 tubes/9 tips
P2	8.1	1.8	1/4	14.0	3 tubes/9 tips
P3	3.5	1.1	2/4	17.0	4 tubes/10 tips
P4	2.1	0.9	0/4	17.0	4 tubes/10 tips
P5	10.4	1.7	1/4	14.0	3 tubes/9 tips
P6	7.6	1.6	2/4	17.5	4 tubes/15 tips

DNA concentration and purity were assessed by Nanodrop spectrophotometry. PCR amplification is expressed as the number of successfully amplified samples over the total tested. Waste volume represents the approximate liquid/plastic waste generated as per the protocol used. Consumables include the total number of tubes and pipette tips required for one extraction.

**FIGURE 4:**
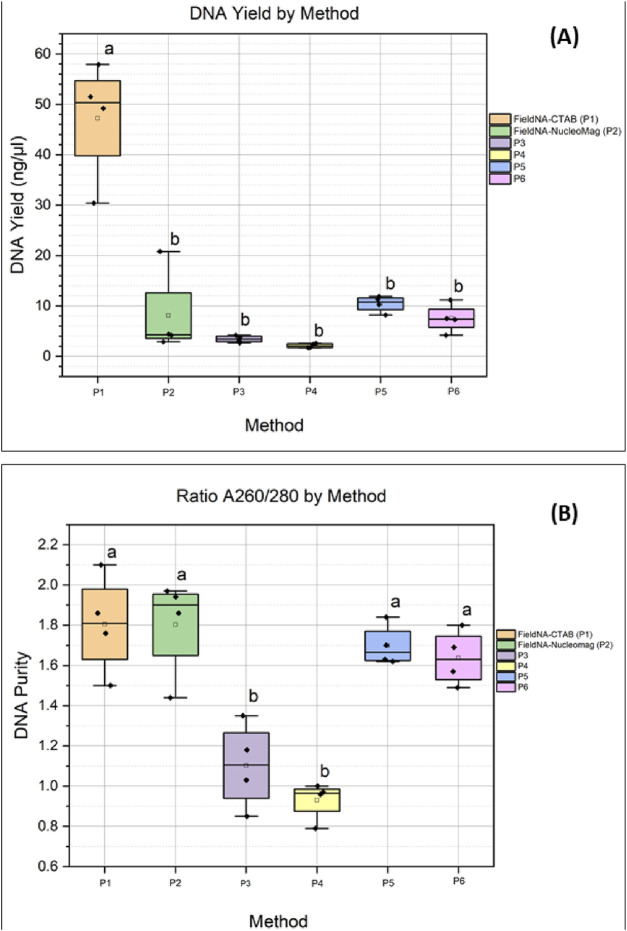
Comparison of the different isolation methods: **(A)** DNA concentration expressed as ng/uL for each method; **(B)** DNA quality measured by absorbance ratio A_260/280_ for each method. Colors indicate the different isolation protocols. Groups sharing a letter are not significantly different by Tukey at α = 0.05; groups with no letters in common are significantly different.

Across the six protocols, DNA yield (in ng/μL) values differed markedly (one-way ANOVA: F (5,18) = 30.97, p = 3.06 × 10^−8^), driven by substantially higher measurements for P1. Tukey’s HSD confirmed that P1 exceeded each of P2–P6 (all p_adj<0.001), while no pairwise differences among P2–P6 were significant after multiplicity correction. In a sensitivity analysis restricted to P2–P6, the omnibus effect was not significant (F (4,15) = 2.82, p = 0.063) and no adjusted pairwise contrasts reached significance, indicating that, apart from P1, the remaining methods performed comparably in this dataset (Supplementary Table S1.2). On the other hand, regarding the ratio A_260/280_, P1 maintained acceptable purity ranting between 1.5 and 2.1, while on the other extreme P4 showed poor purity (0.79–1.00). P6 purity ranged between 1.44 and 1.97, which was comparable with P1, P2 and P5. Overall, from Figure 4B, it is clear that the silica-based spin columns (P3 and P4) provided the poorest purity compared to the rest of the methods. The statistical significance is shown in Figure 4B.

### 3.3 Verification of FieldNA isolated DNA using real-time PCR

The performance of the different DNA isolation protocols was subsequently evaluated using real-time PCR to assess parameters such as cycle values (Cq), PCR efficiency, efficiency R^2^, and melting temperature (T_m_) (Supplementary Table S1.1). It is anticipated that the DNA isolated from the olive leaf samples will exhibit a lower Cq value compared to the DNA isolated from olive oil. This is mainly attributed to the DNA degradation that occurs naturally in the olive oil, since the genomic material is–to its greater degree–stripped and flows freely in the fatty matrix. The main reason for this “free DNA” is the disruption of the olive fruit cells during the crushing (and malaxation step of olive paste), before the olive oil extraction step.

**FIGURE 5:**
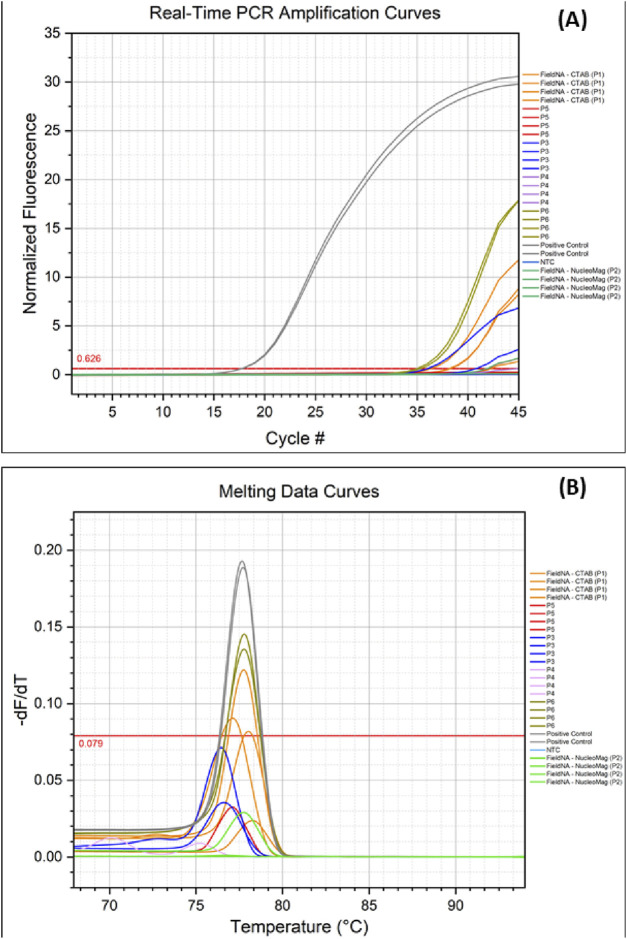
Real-time PCR and Melting analysis: **(A)** Amplification of olive oil DNA isolated with different protocols; **(B)** Melting curve extracted following the real-time PCR experiment, comparing amplified DNA from the different isolation methods. Colors indicate the different methods.

Real-time PCR coupled with High-Resolution Melting (HRM) demonstrated marked performance differences among DNA isolation methods (Figures 5A,B). The positive control (olive leaf gDNA) amplified earliest, as expected, with Cq ≈ 17.75, amplification efficiencies of 0.70–0.74, and excellent linearity (R^2^ > 0.999), confirming assay robustness. Among the different methods of olive oil isolation, P6 yielded the earliest amplification (Cq ≈ 35) with efficiency ≈0.76 and R^2^ > 0.999. P1 showed reproducible amplification at slightly later cycles (Cq 36.3–38.3), efficiencies of 0.73–0.79, and similarly high linearity (R^2^ > 0.999). P5 amplified substantially later (Cq ≈ 41.9), indicative of lower template input despite acceptable efficiency (∼0.79) and high linearity. P3 exhibited variability (Cq 36.0 and 40.8; efficiency 0.72–0.74), consistent with uneven recovery. P4 did not amplify under the conditions tested. No amplification was detected in the no-template control (NTC).

Melting analysis (HRM derivatives) corroborated these results (Figure 5B). The positive control produced a single amplicon with T_m_ = 77.67 °C. P1 (77.61 °C) and P6 (77.76 °C) matched this T_m_ and were the only methods to pass the instrument-defined high-stringency quality filter, supporting specific target amplification. This was further supported by the results of the gel electrophoresis carried out in the PCR amplicons (Supplementary Figure S1.1). P2, P3, and P5 displayed T_m_ values concordant with the expected product but failed the quality filter—consistent with low template abundance and/or suboptimal melt-curve metrics. Overall, P6 provided the most reliable template for downstream real-time PCR, with P1 performing comparably; the remaining kits produced higher Cq values and less robust melt profiles.

## 4 Discussion

DNA isolation from fatty matrices such as olive oil presents significant challenges due to the high content of lipids and phenolic compounds, which can inhibit downstream molecular analyses. Several studies over the years have focused on optimizing the extraction protocols to improve both the DNA yield and its purity, which is essential for applications such as authenticity verification, or detection of admixtures with lower vegetable oils. Raieta et al. (2015) introduced a CTAB-based method combined with hexane and chloroform treatments, that resulted in effectively reduced lipid contamination while it provided DNA of sufficient quality for traceability studies (Raieta et al., 2015). Almost a decade later, Abdelhamid et al. (2024) compared five extraction methods that varied in organic solvents and time-to-results and confirmed that the CTAB-Hexane-Chloroform protocol yielded the highest DNA quality for downstream authentication analysis on Tunisian olive oils (Abdelhamid et al., 2024).

Additional research has been carried out recently with methods that include silica and magnetic bead-based DNA extraction and isolation. These assays have gained attention as efficient alternatives as they require less processing time and, in most cases, do not involve hazardous organic solvents, such as phenol, chloroform and β-mercaptoethanol. Silica-based techniques, such as those adapted from the Boom method, exploit the affinity of DNA for silica particles under chaotropic conditions, followed by binding, washing, and elution steps. Breton et al. (2004) demonstrated the successful application of silica-based extraction for isolating DNA from olive oil, enabling cultivar-specific SSR marker identification (Breton et al., 2004). However, these methods are prone to membrane clogging from oily residues and are sensitive to inhibitors if pre-treatments are inadequate (Doyle and Doyle (1990)). Magnetic bead-based extraction methods employ paramagnetic particles coated with DNA-binding ligands, and offer a faster, scalable, and automation-friendly alternative to silica-bead based platforms, as has been demonstrated by Zhao et al. where this approach was utilized successfully for biomolecules extraction from olive oil (Zhao et al., 2025). However, the cost per extraction for current bead-based DNA extraction methods is higher than that of the aforementioned approaches.

Despite these advancements, several gaps persist in the research. A major limitation is the lack of standardized protocols specifically optimized for olive oil’s unique composition, making cross-study comparisons difficult (Raieta et al., 2015). Moreover, DNA yields remain low and often fragmented (and/or degraded), which limits their use for high-throughput downstream applications (Breton et al., 2004). Effective removal of PCR inhibitors still represents one of the most significant bottlenecks, and while magnetic bead methods are scalable, few validated, automation-compatible workflows for olive oil have been fully developed (Zhao et al., 2025). Indeed, magnetic beads can suffer from binding inefficiencies in cases of very low DNA content and interference from lipids and phenolics, which can lead to suboptimal DNA recovery. There is also a lack of coherent comparative studies across different olive oil types (e.g., extra virgin, refined, flavoured), and pre-treatment steps, such as defatting, are inconsistently applied across the different protocols available in the literature. The above indicate lack of consistency which often requires repetition of experiments and/or combination of multiple molecular markers to authenticate an olive oil and deliver a proper interpretation of DNA testing outcomes, increasing both costs and time-to-results. Thus, the field would greatly benefit from standardized, consistent, efficient, and scalable DNA extraction techniques which are tailored towards the complexities of fatty matrices, ultimately enhancing molecular-based authenticity and traceability in the olive oil industry.

The present study is suitably aimed at demonstrating a more sustainable and cost-effective way of deploying bead-based DNA isolation from EVOO using a novel 3D printed, semi-automated FieldNA device, and verifying its performance against other commercial or widely used DNA isolation/extraction kits/methods. Our findings indicate that when a generic isolation protocol was paired with FieldNA, it resulted in a comparable amplification trend to the more complex, well-established yet laborious CTAB/PCI method. In addition, we also evaluated the device’s performance against the sole commercial kit dedicated for DNA isolation from olive oil, the Norgen olive oil kit. Despite the latter displaying an average DNA yield that was fairly comparable with the second silica-based spin column tested in this study (NucleoSpin Food Kit), it failed in the real-time PCR, which has also been previously observed and reported by Kutateladze et al. (2024) in a study concerning *Helianthus annuus* oil (Kutateladze et al., 2024). Given the results obtained from the herein study using a published SSR marker, we also tested a proprietary primer set (Olea-4) designed by BioCoS that corresponds to a Single Nucleotide Polymorphism (SNP) and the results are presented in Supplementary Figure S1.2. Over the years, many studies have been carried out utilizing SNPs for a diverse panel of organisms and their identification, and it has been widely accepted that these molecular markers can achieve higher resolution in the HRM (Vorimore et al., 2021; Muneeswaran et al., 2024). The reason is mainly attributed to their short amplicon size (usually between 70 bp to a maximum of 150 bp), which makes them better amplification candidates when compared to SSRs that their amplification target is usually larger (above 200 bp).

It is worth considering the FieldNA performance in context of the advantages of device manufacturing process. All major components were 3D-printed allowing for mass production at a large scale. The post-processing step is straightforward, requires minimal labour input, and the magnetic capture module is designed for single use to ensure reproducibility and prevent cross-contamination, enabling a higher throughput of the entire DNA isolation process. It should be noted that UV resin used for 3D printing is generally not biocompatible, preventing a direct DNA isolation on the cartridge surface, however the PVDF film circumvents that limitation and comes pre-fixed to in the cartridge (Macdonald et al., 2016; Brooks and Yadavalli, 2025). This film is also relatively inexpensive and simple to manufacture. In comparison, commercial kits are rather expensive and frequently require external equipment provided by the user whereas the FieldNA system includes all required components.

Another major advantage of this system over commercial kits is its portability. This device can be deployed directly at food production or processing sites, including points along the olive oil supply chain, to enable on-site testing and conduct real time downstream molecular assays using compact, field-deployable instruments. For the current application, in the olive oil industry, this means that regulatory agencies, food authorities, quality control analysts, and olive oil supply chain stakeholders (producers, retailers, importers/exporters, etc.), will be able to conduct on-site DNA testing to quickly screen for fraud (adulteration/admixtures) before sending samples for further analysis in accredited laboratories. By streamlining the initial isolation process on-site, potential fraud can be identified early, reducing overall testing time and enabling faster decision-making regarding the authenticity and integrity of the olive oil. This rapid, field-deployable approach helps prevent compromised food products from reaching consumers and enhances the efficiency of quality control measures.

While the FieldNA device was designed and tested for olive oil DNA isolation, it is fully scalable to other applications. In the current design, the magnetic beads cannot be resuspended after the initial bead capture, which could be a drawback for low DNA yield samples due to the limitations in washing and purification of the captured bead-bound DNA. However, the system’s flexible design allows additional components to be integrated both above and below the device, enabling customization for other specific sample requirements. For example, a heating module and a well for the lysis step can be added vertically above the input module to maintain the gravity-induced passive flow downward. Additionally, a module that facilitates the resuspension of the beads can be incorporated, or the current module can be modified. This flexibility ensures that the system can be expanded with new functionalities and adapted for various applications in both food and health sector, including medical diagnostics.

## 5 Conclusion

In this study we have introduced FieldNA, a novel 3D-printed, gravity-driven device for genomic DNA isolation from olive oil. The prototype demonstrated DNA yields and purity comparable to commercial kits and standard laboratory methods, while offering significant advantages in cost, portability, and reduced consumable use. Its design enables on-site extraction and immediate downstream molecular analysis, providing a practical solution for real-time quality control in the olive oil supply chain. Beyond olive oil, FieldNA is customizable for diverse sample types and can be integrated with compact molecular platforms, including our previously demonstrated portable isothermal RT-PCR system (see Supplementary Figure S1.3; (Staples et al., 2023)), to deliver rapid, sample-to-answer capabilities for food safety applications. With future enhancements such as integrated lysis and temperature control, the device has strong potential to extend its applicability to more complex biological matrices, further broadening its impact as a versatile and sustainable DNA isolation system.

## Data Availability

The original contributions presented in the study are included in the article/Supplementary Material, further inquiries can be directed to the corresponding author.
