# Supplementary material for: FieldNA: a 3D printed vertical microfluidic device for portable nucleic acid isolation from olive oil samples
Source: Front Bioeng Biotechnol. 2025 Oct 13;13:1646041. doi: 10.3389/fbioe.2025.1646041 (PMC12554646; doi:10.3389/fbioe.2025.1646041)
Supplement: Supplementary file 1 [file Supplementaryfile1.docx]

Supplementary Attachment 1

# Supplementary Figures and Tables

## Supplementary Figures


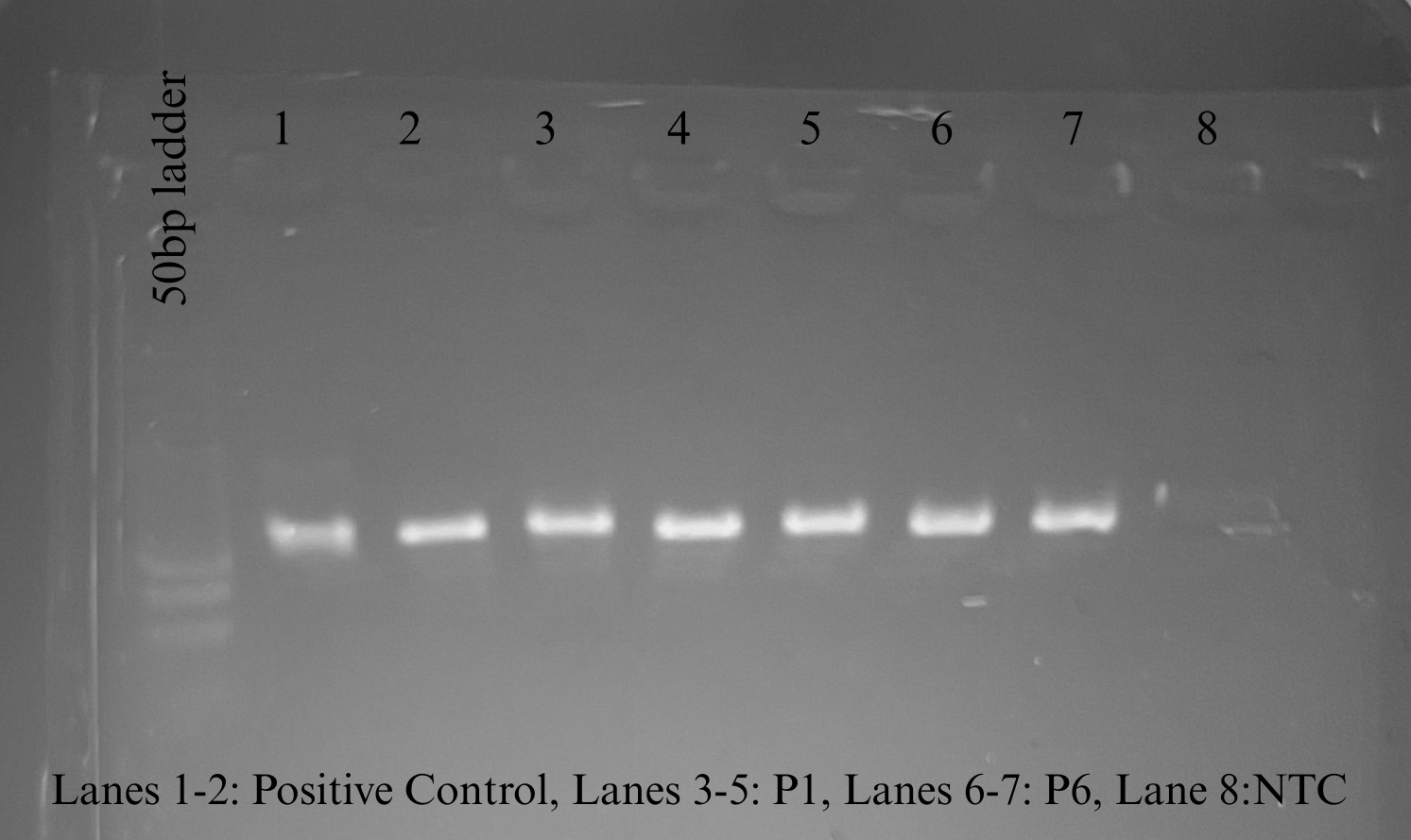


**Supplementary Figure 1.1 2% agarose gel (made with TBE 1x) electrophoresis carried out for the real-time PCR amplicons that passed the Melting Curve threshold. Gel was run at 50V for 60 minutes. Lanes indicate the samples loaded, and a 50bp DNA ladder was utilized.**

**
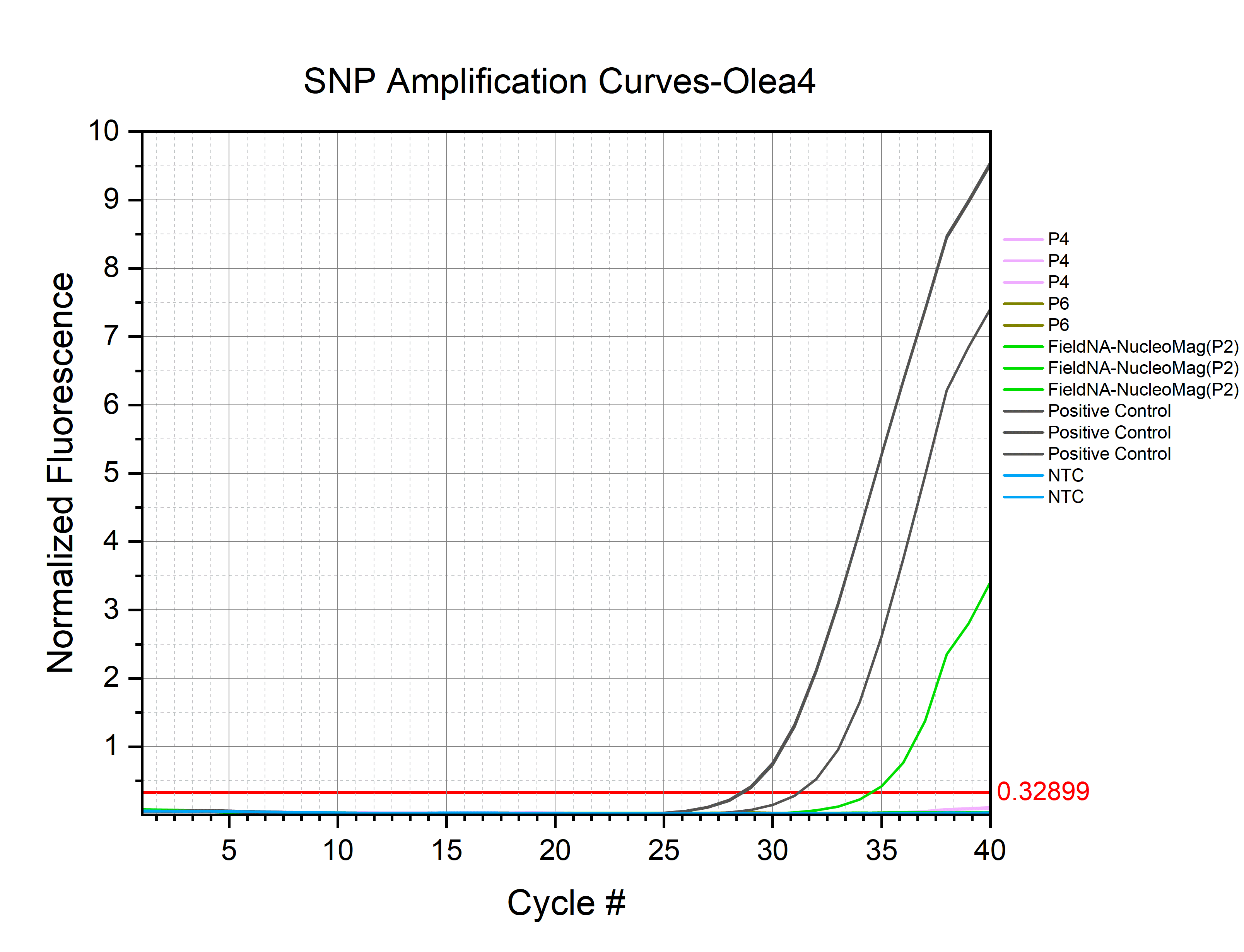
**

**Supplementary Figure 1.2. Performance comparison for the different DNA extraction kits using real-time PCR amplification assays produced using BioCoS’s proprietary SNP (single nucleotide polymorphism) real-time PCR biomarker, Olea4, designed for genotyping EVOO DNA. The result is a promising demonstration of the suitability of the FieldNA device with different downstream molecular assays.**

**
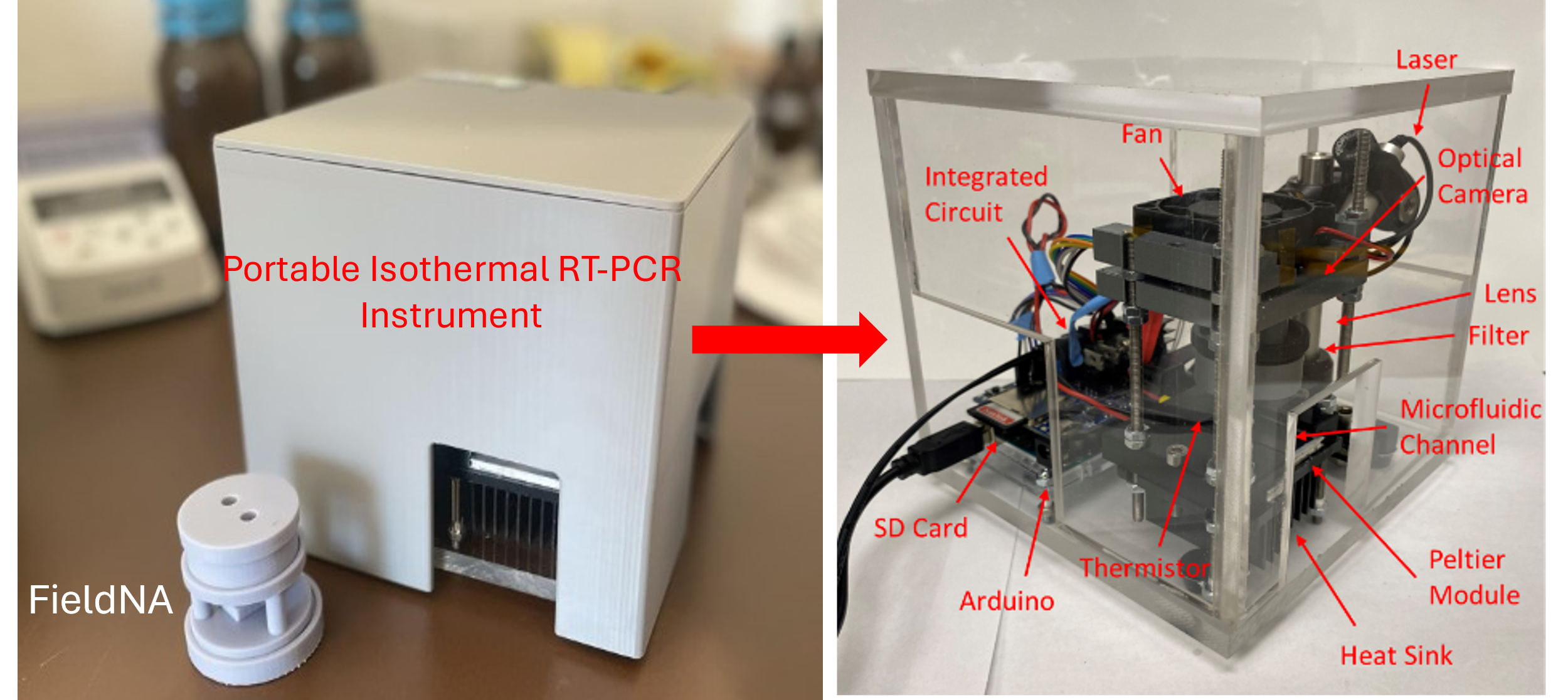
**

**Supplementary Figure 1.3. FieldNA DNA extraction device alongside a portable isothermal RT-PCR platform previously described in Staples et al. (2023).**

## Supplementary Tables

| **Method** | **Cq** | **Efficiency** | **Efficiency R²** | **Result** | **Tm (°C)** |
| --- | --- | --- | --- | --- | --- |
| P1 | 38,26793 | 0,785514 | 0,999994 |  | 77,07 |
|  |  |  |  | Excluded |  |
|  | 36,35146 | 0,73154 | 0,999999 |  | 77,74 |
|  | 38,15083 | 0,757977 | 0,999999 |  | 78,01 |
| P2 |  |  |  | Excluded |  |
|  |  |  |  | Excluded |  |
|  |  |  |  | Excluded |  |
|  | 41,82715 | 0,739321 | 0,999988 |  |  |
| P3 | 36,05044 | 0,735753 | 0,999961 |  |  |
|  |  |  |  | Excluded |  |
|  |  |  |  | Excluded |  |
|  | 40,80983 | 0,720296 | 0,999963 |  |  |
| P4 |  |  |  | Excluded |  |
|  |  |  |  | Excluded |  |
|  |  |  |  | Excluded |  |
|  |  |  |  | Excluded |  |
| P5 | 41,93799 | 0,793505 | 0,999985 |  |  |
|  |  |  |  | Excluded |  |
|  |  |  |  | Excluded |  |
|  |  |  |  | Excluded |  |
| P6 |  |  |  | Excluded |  |
|  |  |  |  | Excluded |  |
|  | 34,99883 | 0,758817 | 0,999997 |  | 77,75 |
|  | 35,3235 | 0,773119 | 0,999994 |  | 77,77 |
| Positive Control | 17,71667 | 0,741359 | 0,999954 |  | 77,64 |
|  | 17,79861 | 0,698545 | 0,999998 |  | 77,7 |
| NTC |  |  |  | Excluded |  |

**Supplementary Table 1.1. Real-time PCR performance metrics for DNA obtained with different isolation protocols. Results include quantification cycle (Cq), amplification efficiency, linearity (Efficiency R²), and melting temperature (Tm). Excluded entries indicate reactions without detectable or reliable amplification. Cq: quantification cycle; Tm: melting temperature. “Excluded” refers to reactions without amplification or with unreliable melt profiles. The positive control (genomic DNA) amplified with low Cq values and uniform melt peaks, whereas the negative control (NTC) showed no amplification.**

| **Contrast (row − col)** | **Mean diff** | **95% CI (lower)** | **95% CI (upper)** | **p_adj** | **Sig.** |
| --- | --- | --- | --- | --- | --- |
| P2 − P1 | −39.175 | −52.8946 | −25.4554 | 5.19×10⁻⁷ | Yes |
| P3 − P1 | −43.800 | −57.5196 | −30.0804 | 9.61×10⁻⁸ | Yes |
| P4 − P1 | −45.125 | −58.8446 | −31.4054 | 6.06×10⁻⁸ | Yes |
| P5 − P1 | −36.825 | −50.5446 | −23.1054 | 1.29×10⁻⁶ | Yes |
| P6 − P1 | −39.700 | −53.4196 | −25.9804 | 4.26×10⁻⁷ | Yes |
| P3 − P2 | −4.625 | −18.3446 | 9.0946 | 0.886 | No |
| P4 − P2 | −5.950 | −19.6696 | 7.7696 | 0.739 | No |
| P5 − P2 | 2.350 | −11.3696 | 16.0696 | 0.993 | No |
| P6 − P2 | −0.525 | −14.2446 | 13.1946 | 1.000 | No |
| P4 − P3 | −1.325 | −15.0446 | 12.3946 | 1.000 | No |
| P5 − P3 | 6.975 | −6.7446 | 20.6946 | 0.599 | No |
| P6 − P3 | 4.100 | −9.6196 | 17.8196 | 0.928 | No |
| P5 − P4 | 8.300 | −5.4196 | 22.0196 | 0.421 | No |
| P6 − P4 | 5.425 | −8.2946 | 19.1446 | 0.804 | No |
| P6 − P5 | −2.875 | −16.5946 | 10.8446 | 0.984 | No |

**Supplementary Table 1.2. One-way ANOVA across P1–P6 was significant, F(5, 18) = 30.97, p = 3.06×10⁻⁸. Tukey’s HSD (α = 0.05; family-wise) pairwise results are shown below. Mean difference is reported as row − column; positive values indicate the row method has a higher mean. “Sig.” marks contrasts that remain significant after Tukey correction.**
