# Supplementary material for: FieldNA: a 3D printed vertical microfluidic device for portable nucleic acid isolation from olive oil samples
Source: Front Bioeng Biotechnol. 2025 Oct 13;13:1646041. doi: 10.3389/fbioe.2025.1646041 (PMC12554646; doi:10.3389/fbioe.2025.1646041)
Supplement: Supplementary file 2 [file Supplementaryfile2.docx]

Supplementary Attachment 2

# Supplementary Figures and Tables

| 1. Photometric comparison of nucleic acid isolation protocols | | | | |
| --- | --- | --- | --- | --- |
| **Protocol** | **Average DNA yield (ng/μl)** | **Consistency (stdev of DNA yield)** | **Average DNA purity A260/280** | **Reproducibility  (stdev of DNA purity)** |
| P2 | 3 | ±1.2 | 1.7 | ±0.2 |
| P4 | 10.9 | ±3.6 | 1.7 | ±0.1 |
| P6 | 6.4 | ±3.7 | 1.6 | ±0.1 |

| 1. **Comparison of Cq values, PCR efficiency and R^2^ values** | | | | |
| --- | --- | --- | --- | --- |
| **Protocol** | **Cq** | **Efficiency** | **Efficiency R²** | **T_m_ (°C)** |
| P2 | 33.13608 | 0.83869 | 0.99962 | 72.04 |
| P4 | 36.37133 | 0.83869 | 0.99962 | ND |
| P6 | 33.71753 | 0.86227 | 0.99984 | 74.33 |
| Positive Control | 22.52138 | 0.91347 | 0.99993 | 78.14 |
| NTC | - | - | - | ND |

**Table 2.1. (A) DNA isolation protocol performance for EVOO-23 samples; (B) Comparison of Cq values, PCR efficiency and R^2^ values for the protocols tested with average of minimum 3 and maximum 4 replicates.**


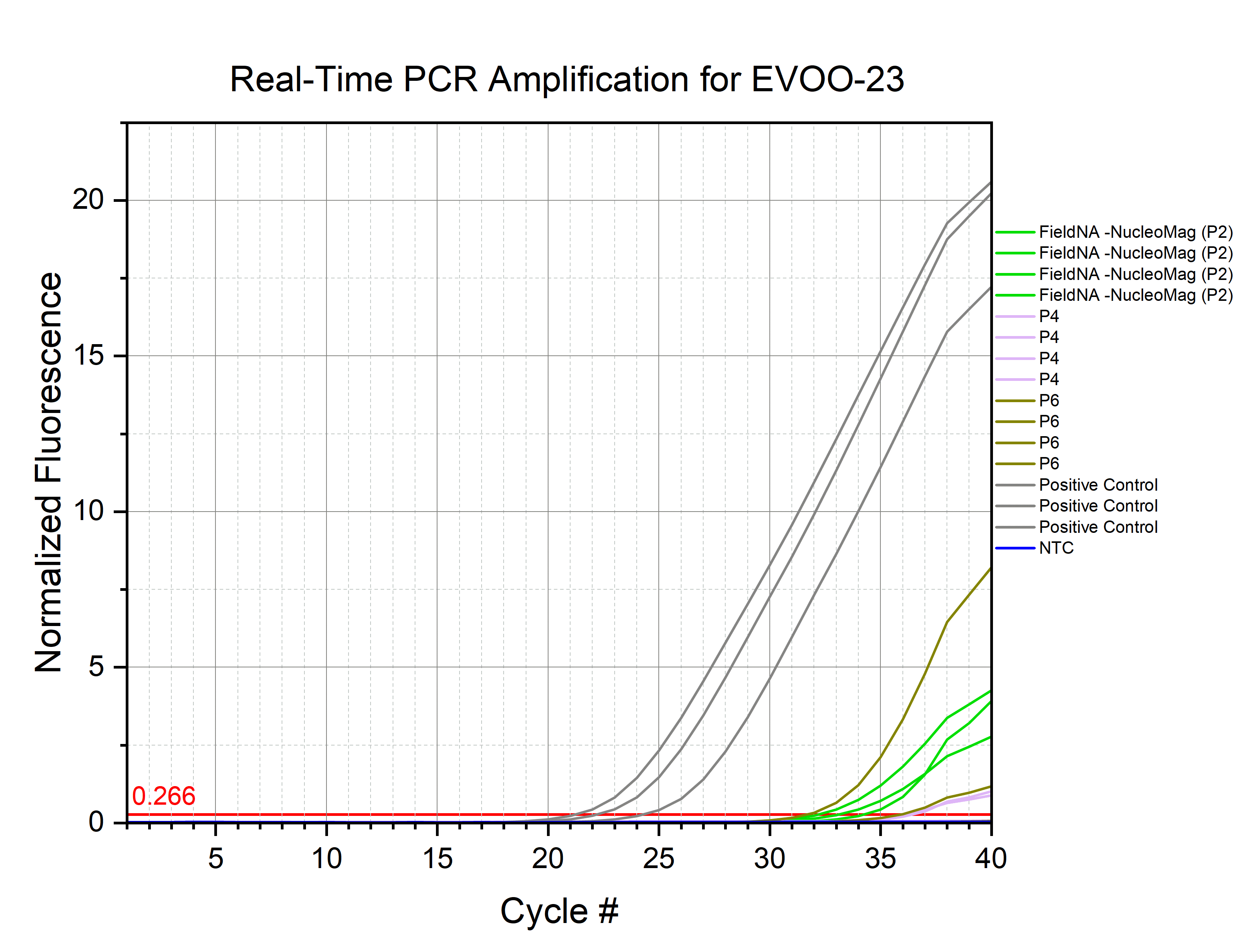


**Supplementary Figure 2.1 Real-time PCR amplification of EVOO-23 olive oil DNA extracted using different protocols. Colors indicate the different extraction methods.**
